# Hematological and Serum Biochemical Changes and Their Prognostic Value in Horses Spontaneously Poisoned by *Crotalaria spectabilis*

**DOI:** 10.3389/fvets.2021.741530

**Published:** 2022-01-14

**Authors:** Antonio Carlos Lopes Câmara, Verônica Lourença de Sousa Argenta, Daniella Dianese Alves de Moraes, Eduardo Ferreira Fonseca, Tayná Cardim Moraes Fino, Giane Regina Paludo, Benito Soto-Blanco

**Affiliations:** ^1^Large Animal Veterinary Teaching Hospital, College of Agronomy and Veterinary Medicine, Universidade de Brasília, Brasília, Brazil; ^2^Secretaria de Estado da Agricultura, Abastecimento e Desenvolvimento Rural Do Distrito Federal, Brasília, Brazil; ^3^Veterinary Clinical Pathology Laboratory, College of Agronomy and Veterinary Medicine, Universidade de Brasília, Brasília, Brazil; ^4^Department of Veterinary Clinics and Surgery, Veterinary College, Universidade Federal de Minas Gerais, Belo Horizonte, Brazil

**Keywords:** direct bilirubin, liver failure, pyrrolizidine alkaloids, gamma-glutamyl transferase, poisonous plants

## Abstract

Determining the prognosis of poisoning by plants containing pyrrolizidine alkaloids is usually challenging. This study aimed to identify important prognostic parameters that can determine the severity of spontaneous poisoning by *Crotalaria spectabilis* in horses. Blood samples from 42 horses spontaneously poisoned by oats contaminated with *C. spectabilis* seeds were evaluated. Complete blood counts (CBC) and serum biochemical tests [urea, creatinine, total protein, albumin, total and direct bilirubin concentrations, aspartate aminotransferase (AST), γ-glutamyl transferase (GGT), and creatine kinase (CK) activities] were performed. Horses were followed up for 12 months to determine the long-term survival rate; after 12 months, they were divided into two groups: survivors (*n* = 30) and non-survivors (*n* = 12). Horses spontaneously poisoned with *C. spectabilis* had higher levels of urea, globulin, bilirubin (total, direct, and indirect), AST, GGT, and CK than the reference values. Non-survivor horses showed significantly higher (*p* < 0.05) values of hemoglobin, GGT, and direct bilirubin than the survivor horses. Horses with serum GGT activity higher than 95 U/l had 14.0 times the risk of death compared to animals showing activities equal to or lower than this value, whereas horses with serum direct bilirubin concentration higher than 0.6 mg/dl (10.26 μmol/L) had 5.78 times the risk of death compared to the others. In summary, serum GGT activity and direct bilirubin concentration may be useful prognostic indicators for assessing the severity of *C. spectabilis*-poisoned horses.

## Background

*Crotalaria* is a plant genus belonging to the Fabaceae family, and it is responsible for the poisoning of horses worldwide (1–5). These plants contain pyrrolizidine alkaloids (PAs), which are bioactivated by the cytochrome P450 system after ingestion, forming toxic pyrrole derivatives. Pyrroles can quickly bind to cellular proteins, DNA, and RNA to form adducts. The main effects of *Crotalaria* poisoning are cellular degeneration and necrosis in the liver, but several *Crotalaria* species also damage the lungs, kidneys, and central nervous system (6, 7).

The clinical signs observed in horses poisoned by *Crotalaria* plants include apathy or depression, ataxia, weight loss, jaundice, aimless wandering, violent behaviors, and proprioceptive deficits (1–3, 5). The main pathological finding in horses with *Crotalaria* poisoning is increased GGT activity due to liver damage (4, 8). Other pathological findings include liver necrosis and fibrosis, megalocytosis, bile stasis, presence of Alzheimer's type II astrocytes in the brain, edema and hemorrhage in the lungs, and degeneration and necrosis of the tubular epithelium of the kidneys (1–3, 5).

Determining the prognosis of poisoning is usually challenging. A study has reported that horses spontaneously poisoned by *Crotalaria medicaginea* showed a death rate of 27.3% when GGT serum activity was higher than 50 U/l, but no death occurred when the activity was below 50 U/l (4). However, for assessing appropriate therapy, it would be beneficial for equine practitioners to have more precise parameters that could indicate whether a horse has an increased risk of death. This study evaluated the hematology and serum biochemistry panel of horses spontaneously poisoned by *C. spectabilis* seeds and determined the most important prognostic parameters.

## Materials and Methods

Ranchers from seven farms located in Midwestern Brazil reported the occurrence of neurological clinical signs in horses. During visits to these farms, the common epidemiological factor was that the oats were from the same producer, and the presence of small dark seeds was verified. After careful manual separation, we found that one kg of oats contained 10 g of seeds compatible with the *Crotalaria* spp. The diagnosis of *C. spectabilis* seed poisoning in horses fed with contaminated oats was established based on the background information, clinical and pathological findings, identification of ingested seeds and adult plants, and measurement of PAs (5). Aflatoxins and fumonisins were measured in the ration and their concentrations ranged from 0 to 0.025 and 1.29 ppm, respectively, which are lower than the minimum concentrations that considered toxic to horses (0.2 ppm for aflatoxins and 10 ppm for fumonisins) (5).

Overall, 42 horses were clinically evaluated from the seven farms. Blood samples were collected by jugular venipuncture into sterile vacuum tubes with ethylenediaminetetraacetic acid (EDTA) and without an anticoagulant. Complete blood counts (CBCs) were performed using a semiautomatic cell counter (Sysmex pocH-100iV *Diff* , Sysmex do Brasil, São Paulo, Brazil). Blood smears were prepared and stained with Diff-Quick for differential leukocyte count and erythrocyte morphological evaluation. Serum concentrations of urea, creatinine, total protein, albumin, total and direct bilirubin and activities of aspartate aminotransferase (AST), γ-glutamyl transferase (GGT), and creatine kinase (CK) were determined using specific commercial kits (Labtest, Lagoa Santa, MG, Brazil) using an semiautomatic analyzer (Bioplus BIO-2000, Barueri, SP, Brazil). Data for total and direct bilirubin concentrations were used to calculate the indirect bilirubin concentrations, and data for total protein and albumin were used to calculate globulins.

The horses (*n* = 42) were followed up for 12 months to determine the long-term survival rate. The 1-year period was stipulated based on previous study that followed-up horses with liver failure from 6 (9) to 14-months (10). Thereafter, horses were distributed into two groups: survivors (*n* = 29) and non-survivors (*n* = 13). The time for death of non-survivor horses are presented at Supplementary Figure 1. Data from hematological and serum biochemistry results were tested for homogeneity of variance using Bartlett's test and were compared using the Mann-Whitney *U*-test. The frequencies of values outside the reference range in hematological and serum biochemical results were compared using Fisher's exact-test for count data. The Odds Ratio and Risk Ratio of death was determined in horses with serum GGT activity ≥ 95 U/l and serum direct bilirubin concentration ≥ 0.6 mg/dl using Epi Info v.7.2.4.0. The statistical significance level was set at *p* < 0.05.

## Results

Table 1 shows that the hemoglobin values of non-survivor horses were significantly higher (*p* < 0.05) than those of the survivor horses but that the values were within the reference range. Individual values of red blood cells, hemoglobin, mean corpuscular hemoglobin concentration, white blood cells (WBC), neutrophils, band neutrophils, lymphocytes, monocytes, and platelets were found to be beyond the reference range for both the groups. However, only the changes in WBC numbers were significantly different between the two groups (Table 2).

**Table 1:** Complete blood counts and fibrinogen of horses spontaneously poisoned by *Crotalaria spectabilis* seeds, that survived (*n* = 29) or not (*n* = 13) for up to 12 months.

	**Group**		
**Parameter**	**Survivors**	**Non-survivors**	**Reference range^a^**
PCV (%)	39.0 (29) 24–47	40 (12) 24–53	24–53
RBC (× 106/μl)	8.65 (29) 4.84–12.6	8.95 (12) 5.06–10.9	5.5–12.9
Hemoglobin (g/dl)	13.0 (29) 9.10–18.2	14.1 (12)^*^ 9.10–19.7	8.0–19.0
MCV (fl)	46.0 (29) 40–55	48.5 (12) 35–53	37–58.5
MCHC (%)	33.0 (29) 29–40	34.0 (12) 31–39	31.0–38.5
WBC (× 103/μl)	9.60 (29) 5.40–12.2	8.75 (12) 6.90–44.5	5.0–14.3
Neutrophil (/μl)	5,442.5 (26) 2,106–9,576	5,800 (11) 4,108–38,715	2,260–8,580
Band neutrophil (/μl)	0 (26) 0–0	0 (11) 0–1,335	0–100
Lymphocyte (/μl)	3,258 (26) 612–5,280	2,670 (11) 350–9,984	1,500–7,700
Monocyte (/μl)	194 (26) 0–515	222 (11) 0–1780	0–1,000
Eosinophil (/μl)	277.5 (26) 0–826	79 (11) 0–814	0–1,000
Basophil (/μl)	0 (26) 0–136	0 (11) 0–156	0–290
Fibrinogen (mg/dl)	200 (12) 100–400	200 (11) 100–1,000	100–400
Platelets (× 103/μl)	137.0 (27) 47.0–279.0	107.5 (12) 26.0–184.0	100–350

*Poisonings occurred in Distrito Federal and Goiás state, Midwestern Brazil, in November 2018-March 2019. Data are presented as median (n) and minimum–maximum*.

a *Kramer (11)*.

* *Significantly different from recovery group (p < 0.05, Mann-Whitney-test)*. *PCV, packed cell volume; RBC, red blood cell; MCV, mean corpuscular volume; MCHC, mean corpuscular hemoglobin concentration; WBC, white blood cell*.

**Table 2:** Frequencies of individual values outside the reference range in complete blood counts of horses spontaneously poisoned by *Crotalaria spectabilis* seeds, that survived (*n* = 29) or not (*n* = 13) for up to 12 months.

**Parameter**	**Group**	
	**Survivors**	**Non-survivors**
RBC	Decreased in 1/29 (3.45%)	Decreased in 1/12 (8.33%)
Hemoglobin	No change in 29	Increased in 1/12 (8.33%)
MCHC	Decreased in 2/29 (6.90%) Increased in 1/29 (3.45%)	Decreased in 1/12 (8.33%)
WBC	No change in 29	Increased in 4/12 (33.3%)^*^
Neutrophil	Decreased in 1/26 (3.85%) Increased in 3/26 (11.5%)	Increased in 2/11 (18.2%)
Band neutrophil	No change in 26	Increased in 1/11 (9.09%)
Lymphocyte	Decreased in 5/26 (19.2%)	Decreased in 5/11 (45.5%) Increased in 1/11 (9.09%)
Monocyte	No change in 26	Increased in 1/11 (9.09%)
Platelets	Decreased in 6/27 (22.2%)	Decreased in 5/12 (41.7%)
Fibrinogen	No change in 12	Increased in 1/11 (9.09%)

*Poisonings occurred in Distrito Federal and Goiás state, Midwestern Brazil, in November 2018-March 2019*.

*RBC, red blood cell; MCHC, mean corpuscular hemoglobin concentration; WBC, white blood cell*.

* *Significantly different from recovery group (p < 0.05, Fisher's Exact-Test for Count Data)*.

In the serum biochemical panel (Table 3), non-survivor horses had significantly higher (*p* < 0.05) values of AST, GGT, and direct bilirubin than the survivor horses. The individual values of all evaluated parameters were beyond the reference range; however, significantly different ratio of values out of the reference range between the two groups were found only for urea concentration (Table 4).

**Table 3:** Serum biochemical panel of horses spontaneously poisoned by *Crotalaria spectabilis* seeds, that survived (*n* = 29) or not (*n* = 13) for up to 12 months.

**Parameter**	**Group**		**Group**
	**Survivors**	**Non-survivors**	**Reference range^a^**
Urea (mg/dl)	33.0 (23) 15.0–83.0	19.0 (12) 14.0–100.0	10–24
Creatinine (mg/dl)	1.60 (22) 0.80–2.20	1.40 (10) 0.72–1.70	1.2–1.9
Total proteins (g/dl)	7.30 (17) 6.47–8.70	6.75 (10) 4.30–8.90	5.2–7.9
Albumin (g/dl)	2.85 (17) 1.88–3.40	2.25 (10) 1.68–2.90	2.6–3.7
Globulins (g/dl)	4.51 (17) 3.56–6.60	4.52 (10) 2.62–6.40	2.6–4.0
AST (UI/L)	374.2 (25) 54.8–973.3	474 (11)^*^ 290–2,350	226–366
GGT (UI/L)	66.0 (26) 30.0–297.5	288 (13)^*^ 92–600	0–11.8
CK (UI/L)	434.5 (24) 144.2–3,281.7	393.7 (8) 194–3,400	<140
Total bilirubin (mg/dl)	1.51 (25) 0.40–5.35	4.90 (7) 0.90–25.6	1.0–2.0
Direct bilirubin (mg/dl)	0.48 (25) 0.20–0.75	0.70 (7)^*^ 0.30–10.1	0–0.4
Indirect bilirubin (mg/dl)	1.03 (25) 0.10–5.01	4.10 (7) 0.40–15.5	0.2–2.0

*Data are presented as median (n) and minimum–maximum. Poisonings occurred in Distrito Fe zderal and Goiás state, Midwestern Brazil, in November 2018-March 2019*.

a *González and Silva (12)*.

* *Significantly different from recovery group (p < 0.05, Mann-Whitney-test)*. *AST, aspartate aminotransferase; GGT, γ-glutamyl transferase; CK, creatine kinase*.

**Table 4:** Frequencies of individual values outside the reference range in serum biochemical panel of horses spontaneously poisoned by *Crotalaria spectabilis* seeds, that survived (*n* = 29) or not (*n* = 13) for up to 12 months.

**Parameter**	**Group**	
	**Survivors**	**Non-survivors**
Urea	Increased in 18/23 (78.3%)	Increased in 4/12 (33.3%)^*^
Creatinine	Decreased in 3/26 (11.5%) Increased in 4/26 (15.4%)	Decreased in 4/10 (40.0%)
AST	Increased in 15/25 (60%)	Increased in 10/11 (90.9%)
GGT	Increased in 26/26 (100%)	Increased in 13/13 (100%)
Total proteins	Increased in 5/7 (29.4%)	Decreased in 1/10 (10.0%) Increased in 3/10 (30.0%)
Albumin	Decreased in 7/17 (41.2%)	Decreased in 6/10 (60.0%)
Globulins	Increased in 13/17 (76.5%)	Increased in 8/10 (80.0%)
Creatine kinase	Increased in 24/24 (100%)	Increased in 8/8 (100%)
Total bilirubin	Decreased in 5/25 (20.0%) Increased in 8/25 (32.0%)	Decreased in 1/7 (14.3%) Increased in 4/7 (57.1%)
Direct bilirubin	Increased in 15/25 (60.0%)	Increased in 6/7 (85.7%)
Indirect bilirubin	Decreased in 2/25 (8.0%) Increased in 6/25 (24.0%)	Increased in 4/7 (57.1%)

*Poisonings occurred in Distrito Federal and Goiás state, Midwestern Brazil, in November 2018-March 2019*.

*AST, aspartate aminotransferase; GGT, γ-glutamyl transferase*.

* *Significantly different from recovery group (p < 0.05, Fisher's Exact-Test for Count Data)*.

For serum GGT activity higher than 95 U/l, the risk ratio was 14.0 (2.02–97.5) and the risk difference was 61.9% (38.3–85.5%) (Supplementary Table 1). *P*-value using Mantel-Haenszel test was <0.0001.

For serum direct bilirubin concentration higher than 0.6 mg/dl (10.26 μmol/l), the risk ratio was 5.78 (1.73–19.3) and the risk difference was 55.1% (15.5–94.8%) (Supplementary Table 2). *P-*value using Mantel-Haenszel test was 0.0038.

## Discussion

This study assessed the hematological and serum biochemical changes in horses poisoned by *C. spectabilis*. In 28% of poisoned horses, there was a decrease in the number of platelets. Monocrotaline-induced reduction in the circulating number of platelets was described earlier in rodents (13, 14) because it induces blood clotting (14, 15) and platelet aggregation (16). This hemostasis dysfunction can obstruct the sinusoidal vessels and cause liver damage (14–16).

The number of WBC (leukocytosis) increased in 33% of the non-survivor horses, whereas no change was found in survivor horses. Differential leukocyte counts showed that 13.5% of the poisoned horses had increased neutrophils (neutrophilia) and that 27% of the horses had reduced lymphocytes (lymphopenia), but no significant difference was found between the survivor and non-survivor groups. These hematological changes indicate an inflammatory process (11, 17). Monocrotaline induces a hepatic inflammatory response that enhances liver injury (18, 19). Neutrophilia is caused by the release of neutrophils from storage and maturation pools of the bone marrow to the circulation in response to inflammatory cytokines and to adrenalin realize during acute stress (11, 17). Immunotoxic effects of monocrotaline can cause the death of lymphocytes, leading to reduced levels of circulating lymphocytes. Studies have shown that administration of monocrotaline in female mice reduced the thymus and spleen weights and cellularity, and also impaired the antibody-mediated immunity and T cell-mediated cytotoxicity (20, 21). Another reported effect was the suppression of the blastogenic response to B and T cell mitogens (21, 22). It is also important to note that microscopic evaluation of livers from *Crotalaria*-poisoned horses showed lymphohistiocytic inflammatory infiltrate (5).

Another hematological finding was that hemoglobin concentrations were significantly higher in non-survivor horses (*p* < 0.05) than in survivor horses. However, this difference needs further validation because the concentrations were within the reference range, and the concentration in only one horse in the non-survivor group was above the reference range, which could be due to dehydration (23).

Results of serum biochemical panel (Table 3) showed that most of the evaluated parameters were affected by *C. spectabilis* poisoning. Elevated bilirubin levels and AST and GGT activities may be associated with liver damage (23–25), which is the major toxic effect of *Crotalaria* (2–4) and other pyrrolizidine alkaloid-containing plants (26–31). The poisoned horses showed higher levels of globulins and decreased albumin, both of which indicate hepatic disease (32). In fact, a total of 13 horses poisoned by *C. spectabilis* were necropsied and confirmed hepatic necrosis and fibrosis (5). Serum urea and creatinine are linked to impaired renal functions (23), and several *Crotalaria* species induce degeneration and necrosis of the tubular epithelium (2, 4), which as also observed in the horses from the present study (5). Elevated serum urea levels were more frequent in the survivor than in the non-survivor horses, which could be due to the impaired conversion of ammonia into urea as a consequence of liver damage (32). The microscopic evaluation of the brains of *Crotalaria*-poisoned horses suggested the occurrence of hepatic encephalopathy due to elevated serum ammonia levels (5). Increased serum CK activity occurs in cases of muscle damage (23, 25) and this increase may be intensified in recumbency cases. CK activity can also increase due to hyperbilirubinemia, which leads to a false-positive result (23). In the present study horses with serum GGT activity higher than 95 U/l had 14.0 times the risk of death compared to animals showing activities equal to or lower than this value. GGT is a sensitive and specific serum marker of liver disease in horses, and increased serum activity is observed in cases of cholestasis, biliary necrosis, or hyperplasia (23–25). Other authors have also verified that serum GGT activity is useful for monitoring horses poisoned by PAs (26–31). Additionally, measurement of serum GGT activity was useful for differentiating horses with subclinical *Crotalaria* spp. poisoning from the unaffected horses (8). After spontaneous poisoning by *C. medicaginea*, 12 of the 44 (27.3%) horses with GGT activity higher than 50 U/l died during the study, whereas all the 14 horses with GGT activity lower than 50 U/l survived (4). Furthermore, when evaluating primary hepatitis, hepatic lipidosis, and cholelithiasis in horses and donkeys, the cut-off value for GGT as a predictor of fatal outcome was 224 U/l, which gave maximal values of 54% for sensitivity and 95% for specificity (32).

In this study, horses with serum direct bilirubin concentration higher than 0.6 mg/dl (10.26 μmol/L) had 5.78 times the risk of death compared to the others. Liver failure in horses is usually associated with an increased serum direct bilirubin concentration, and this parameter is an important indicator of liver damage (25). Indirect bilirubin levels were also increased in the poisoned horses and may have been caused by other conditions, especially anorexia (25). In horses, hepatic diseases associated with severe or irreversible liver damage, such as pyrrolizidine alkaloid toxicity, present permanent or progressive loss of hepatic function (2, 26, 29). Serum direct bilirubin in combination with GGT activity levels can be used as prognostic indicators to assess the severity of poisoning by pyrrolizidine alkaloid-containing plants in horses, especially because increases in these two parameters are related to lesions in the biliary canaliculus (23, 24).

## Conclusions

Horses spontaneously poisoned by *C. spectabilis* seeds showed leukocytosis, lymphopenia, thrombocytopenia, and increased serum urea, creatinine, globulins, bilirubin (total, direct, and indirect), AST, GGT, and CK. Serum GGT activity (≥95 U/l) levels and serum direct bilirubin concentration (≥0.6 mg/dl) may be useful prognostic indicators for determining the severity of *C. spectabilis* poisoning in horses.

## Data Availability Statement

The raw data supporting the conclusions of this article will be made available by the authors, without undue reservation.

## Ethics Statement

Ethical review and approval was not required for the animal study because this paper serves to describe the laboratory findings related to spontaneous poisoning by *Crotalaria spectabilis* in horses. Management of the cases was not altered by the study and no ethical approval was obtained. Written consent was obtained from the owners for laboratory exams. Written informed consent was obtained from the owners for the participation of their animals in this study.

## Author Contributions

AC, VS, DM, EF, TF, and GP performed clinical and laboratory examinations. BS-B performed the statistical analysis. GP, BS-B, and AC drafted the manuscript. All authors critically revised the manuscript and gave final approval.

## Funding

This study was partially supported by the Conselho Nacional de Desenvolvimento Científico e Tecnológico–Brazil (CNPQ) (Process 311182/2017-8).

## Conflict of Interest

The authors declare that the research was conducted in the absence of any commercial or financial relationships that could be construed as a potential conflict of interest.

## Publisher's Note

All claims expressed in this article are solely those of the authors and do not necessarily represent those of their affiliated organizations, or those of the publisher, the editors and the reviewers. Any product that may be evaluated in this article, or claim that may be made by its manufacturer, is not guaranteed or endorsed by the publisher.
