# Supplementary material for: Hematological and Serum Biochemical Changes and Their Prognostic Value in Horses Spontaneously Poisoned by Crotalaria spectabilis
Source: Front Vet Sci. 2022 Jan 14;8:741530. doi: 10.3389/fvets.2021.741530 (PMC8795585; doi:10.3389/fvets.2021.741530)
Supplement: Supplementary file 1 [file Data_Sheet_1.docx]

Figure S1. Time (in weeks) for for death of horses spontaneously poisoned by *Crotalaria spectabilis* seeds.


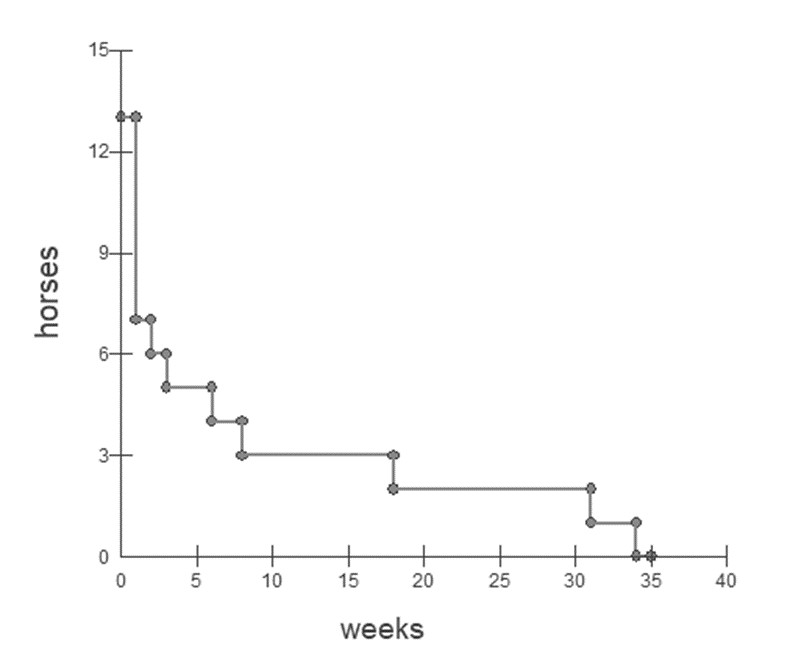


Table S1: Predictive value (odds ratio and risk ratio) of serum γ-glutamyl transferase (GGT) activity for death within 12 months in horses spontaneously poisoned by *Crotalaria spectabilis* seeds (n=39) using Epi Info v.7.2.4.0.

| **Parameter** | **Value** |
| --- | --- |
| Lethality > 95 U/L | 66.7% (12 of 18) |
| Lethality ≤ 95 U/L | 4.76% (1 of 21) |
| Odds ratio | 40.0 (4.28 – 373.8) |
| MLE odds ratio | 35.4 (4.82 – 898.7) |
| Risk ratio | 14.0 (2.02 – 97.5) |
| Risk difference (%) | 61.9 (38.3 – 85.5) |
| P (Mantel-Haenszel test) | <0.0001 |
| P (Fisher Exact test) | <0.0001 |

Table S2: Predictive value (odds ratio and risk ratio) of serum direct bilirubin concentration for death within 12 months in horses spontaneously poisoned by *Crotalaria spectabilis* seeds (n=32) using Epi Info v.7.2.4.0.

| **Parameter** | **Value** |
| --- | --- |
| Lethality > 0.6 mg/dl | 66.7% (4 of 6) |
| Lethality ≤ 0.6 mg/dl | 11.5% (3 of 26) |
| Odds ratio | 15.3 (1.91 – 122.8) |
| MLE odds ratio | 13.3 (1.70 – 144.4) |
| Risk ratio | 5.78 (1.73 – 19.3) |
| Risk difference (%) | 55.1 (15.5 – 94.8) |
| P (Mantel-Haenszel test) | 0.0038 |
| P (Fisher Exact test) | 0.0121 |
